# Incidental learning of group trust: Predictive gaze cue matters

**DOI:** 10.1038/s41598-020-64719-5

**Published:** 2020-05-08

**Authors:** Zhongqiang Sun, Zhihui He, Guochao Zhang, Xinyu Li, Wenjun Yu

**Affiliations:** 1 Department of Psychology, Ningbo University, Ningbo, 315211 China; 2 Center of Group Behavior and Social Psychological Service, Ningbo University, Ningbo, 315211 China; 3 Department of Psychology, Zhejiang Normal University, Jinhua, 321004 China; 4 Business School, Ningbo University, Ningbo, 315211 China; 5 Academy of Neuroeconomics and Neuromanagement, Ningbo University, Ningbo, 315211 China

**Keywords:** Psychology, Human behaviour

## Abstract

Human gaze is a subtle cue to deliver information and helps impression formation in social interactions. People automatically follow the gaze direction of others and shift their attention accordingly, as well as determine the trustworthiness of others based on the predictable validity of their gaze behavior, yet it remains unclear how this works at the collective level. Therefore, the current study is the first to explore the incidental learning of trust from a group’s gaze behavior. To simulate different patterns of perceiving collective information in real life, two ways of presenting group member gazes were used in the object categorization task, the simultaneous way in Experiment 1 and the sequential way in Experiment 3, and a sampling strategy was ruled out in Experiment 2. Converging findings in experiments demonstrated a typical gaze-cueing effect, and more importantly, the Predictive-valid group obtained more trust compared to the Predictive-invalid group. To enrich and expand the applicability of the incidental trust learning effect from gazes, the current study provides supportive evidence at the collective level, confirming that humans have an efficient capability to process gaze information of groups.

## Introduction

Social interactions happen every day between individuals or groups. It is important to make quick evaluations and form appropriate impressions of other people, which would benefit both sides’ future communication and cooperation. Accordingly, an important objective in the field of social cognition is to understand the impression formation process on the basis of various cues, including both explicit and subtle ones^1,2^. As a subtle cue, human gaze delivers information and helps impression formation in social interactions^3,4^. When we observe an individual looking at a particular position, we automatically follow this gaze direction and shift attention. Objects in that gazed-at location are found to be processed faster and more accurately than those not cued in other places, which is named the gaze-cueing effect^5,6^. Previous studies have shown that this effect is strong and difficult to eliminate. That is, people follow the gaze direction even if the gaze cue is emphasized as uninformative^7,8^.

Many downstream effects on cognition can be triggered by this attention-following behavior. Objects that are gazed at are remembered better^9,10^ and liked more than ignored ones^11–13^. The gaze-cueing effect not only impacts processing of related targets but also helps the imperceptible impression formation of the cueing face. In the initial investigation of incidental learning of trust from gaze, Bayliss and Tipper^14^ modified a gaze-cueing paradigm such that some individuals’ gaze was always helpful and directed towards the place where the target would subsequently appear (valid cue); contrariwise, other individuals gazed where nothing appears (invalid cue), which is deceptive. Participants were then shown pairs of faces (one helpful, one deceptive) and asked to choose the more trustworthy one. They frequently selected the face with helpful gaze, even though they had been previously instructed that the gaze was irrelevant to targets and should be ignored. In this case, observers unconsciously extracted the internal state of the cueing person from the gaze validity and made a further inference about the cueing person’s trait trustworthiness. This subtle effect of gaze has been frequently replicated and extended by numbers of studies in the recent decade. For instance, researchers indicated a good persistence of this incidental trust from gaze behaviors that lasted for approximately one hour^15^. Moreover, facial physiological features were revealed to manipulate the incidental learning of trust from gaze cue. Bayliss and colleagues^16^ found that trust learning occurs when the gazes have positive social connotations such as smiling, rather than a negative emotional expression. Concerning racial distinctions, gaze behavior from other-race faces is of no help in gaining trust^17^. Moreover, recent studies have begun investigating the underlying mechanism of trust-learning with neuroscientific techniques^18,19^. Electromyographic studies have shown that embodied emotional reactions to gaze cues mediate trust learning^18^.

Obviously, social interaction between two individuals is not the only case in real life. Trust has a great effect on all kinds of social exchange, ranging from goods to services and from the individual to the collective level. We also make perception and trustworthiness judgments of certain groups of people. This is because many important decisions are made on different scales of collective action rather than the individual level, including small groups like the family and large groups like governments or firms^20,21^. Studies have confirmed that the sense of trust is as an important component of social impression^22^, yet, as previous studies mostly investigated trust learning at individual level, little work has been done in the context of collective trust learning. Thus, it remains unclear whether this incidental trust learning by gaze could happen for groups of people.

Regarding trust learning from group gazes, existing findings provide inconsistent evidence for incompatible predictions. On one hand, a review of impression formation highlighted the fundamental distinctions between individual and collective impression formation^23^, suggesting that participants might perceive a higher level of homogeneity from individuals than groups, leading to more frequent and easier inferences about individuals than groups. Subsequent studies have accumulated evidence for such a difference between single and group information^24,25^. Moreover, the sense of trust may not be gained based on nuanced group gaze behavior due to a lack of cognitive resources. Based on previous findings that extra cognitive resources are required to retain multi-feature bindings^26,27^, we speculated that there is analogously an increased recruitment of cognitive resources in binding multi-gaze behaviors and face identity. At the same time, considering the possibility that people would not voluntarily invoke cognitive resources without a clear task requirement, group perception and information maintenance may be inferred to be due to a lack of cognitive resources, resulting in the failure on trust learning from group gazes. Additionally, literature suggests that humans interact more frequently with other individuals than with groups in real life scenarios^28^. Compared to group behaviors, the greater frequency of observations of individual behaviors helps the observer to develop a more routinized inference process on individual impression formation. Taken together, these results suggest that trust learning from group gazes cannot simply parallel to individual gazes.

On the other hand, numerous studies have shown that humans are able to perceive and integrate social information of a group of people^29^. Observers are capable of extracting perceptual ensemble representations of group attributes including facial expression^30^, race^31^ and gender^32^. Importantly, recent advances in gaze perception have shown that the gaze direction of a collective can be efficiently perceived^33,34^, followed by a rapid attention shift to where a group of people consistently gaze as would occur in response to a single gazer^35–37^. This following effect is in fact amplified by increasing numbers of consistently directed gazes^38,39^. This fact supports a different prediction that group gaze information might be proficiently processed to generate a group impression.

Given these two competing possibilities, it is first necessary to explore whether humans are able to gain a sense of trust toward a group from group gaze behaviors. To simulate two ways of perceiving group information in real life, experiments with different manipulations of how the collective was presented were conducted. Using a gaze-cueing paradigm in an object categorization task, Experiment 1 presented all faces in a collective simultaneously^28,37^, while a potential sampling strategy was tested in Experiment 2; Experiment 3 sequentially presented them with a preset group name below each face image^40^. The second part of all three experiments was a surprise face-choice task having the same purpose of picking out faces that gained more trust in the previous object categorization task.

## Experiment 1

### Methods

#### Participants

Thirty graduate and undergraduate students (17 females; mean age 20.21 years) were paid to participate in this experiment. All participants had normal or corrected-to-normal vision, and provided their written informed consent in accordance with the Declaration of Helsinki before the experiment.

The sample size of the study was determined via a power analysis using G*Power 3^41^. Given an alpha level of 0.05 and a power of 0.8 to detect a large effect size (*f* = 0.50), the power analysis suggested a sample size of 27. Considering necessary exclusions, we decided to stop collecting data at *N* = 30. One participant reported that she was aware of gaze predictability for targets and made a correct guess as to the experimental objective, so we excluded her data, leaving the data of 29 participants for further analysis.

#### Stimuli

All face stimuli were taken from the SCUT-FBP5500 database^42^. Twelve independent raters were invited to ensure approximately equal facial trustworthiness and attractiveness across conditions, resulting in 24 male and 24 female Asian faces with the same approximate age as the cue stimuli. Faces were divided into 16 groups of three faces each. The three faces in each group were always the same gender and positioned in one row. Three versions of each group were produced using ADOBE PHOTOSHOP CS6, one with all three faces gazing straight ahead, one with gazes averted leftward, and another with gazes averted rightward. Each of the three faces measured 2° × 2.8° and were clearly displayed, not being covered by any other face. These groups of faces were arranged so as to constitute two conditions and presented as either predictive-valid or predictive-invalid cues in the experiment (counterbalanced among participants).

The target stimuli consisted of 20 items, half of which were office supplies and the rest were kitchen supplies. Each item measured approximately 1.8° × 1.8°, centered 5° to the left or right of the screen center. All stimuli were presented on a gray background (RGB, 80, 80, 80).

## Procedure and design

Participants were seated at a distance of 70 cm from a CRT monitor (17-inch, 100-Hz refresh rate). PRESENTATION software was used to present stimuli and acquire responses. Two main tasks were sequentially arranged in the experiment. At the beginning of the experiment, participants were told to perform the object categorization task. After the first task finished, they were told the second task. The experimenter gave clear instructions on how to perform the trials. The study designs and experimental procedures in Experiments 1, 2, and 3 were all approved by the Research Ethics Board of Department of Psychology at Ningbo University.

### Object categorization task

**Figure 1:**
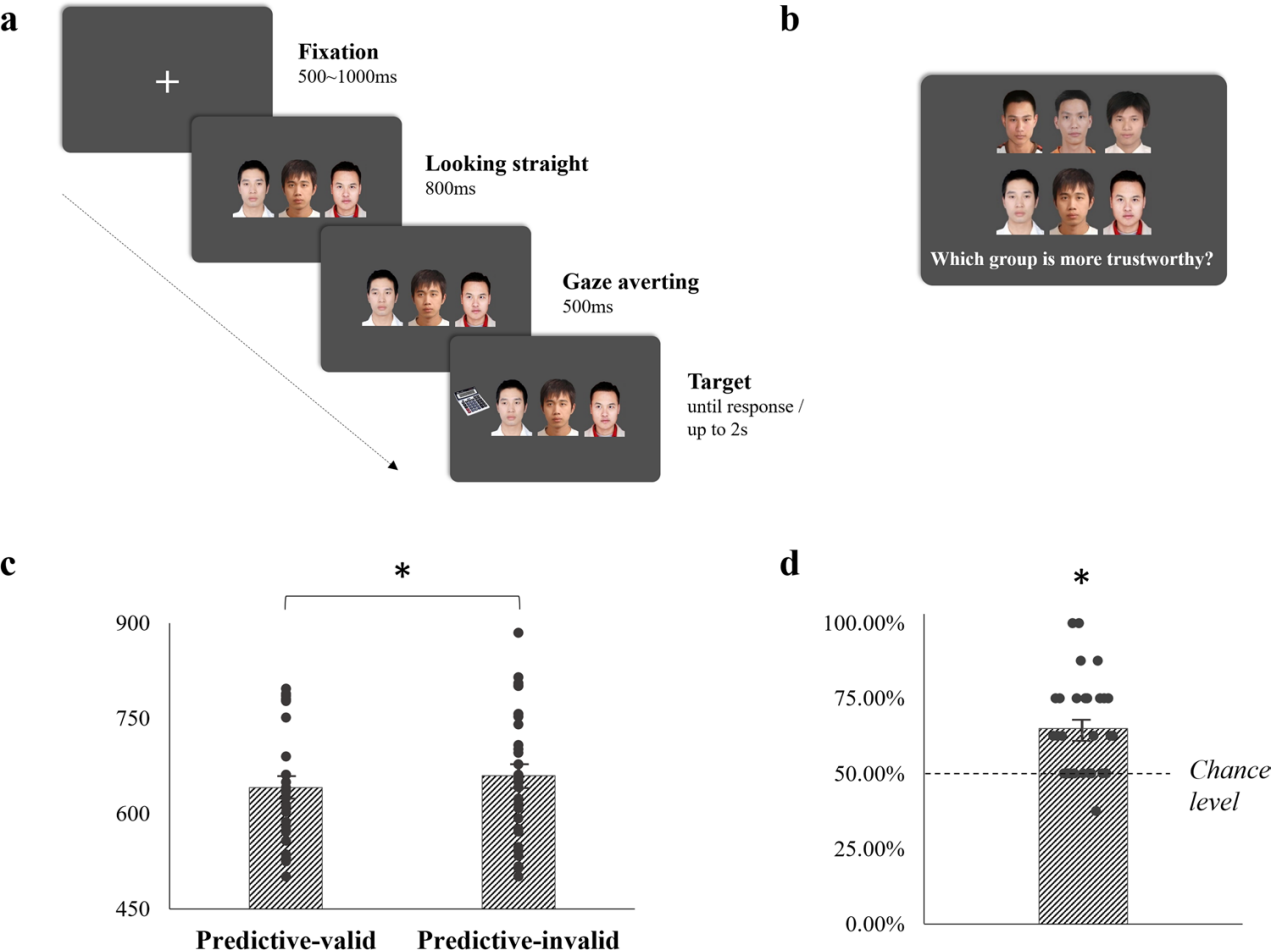
Procedure and results in Experiment 1. (**a**) An example of the trial in the object categorization task with the Predictive-valid group gazes and an office item as the target; (**b**) an example of the trial in the face-choice task; (**c**) RT results in the object categorization task; (**d**) results of percentage choosing the Predictive-valid group as more trustworthy, and the dashed line represents the 50% chance level. The dots in (**c**) and (**d**) are individual data points making up each group. The asterisk represents a significant difference between two corresponding conditions or from the chance level (*p* < 0.05), and the error bar represents one S.E.M.

The first part of experiment was an object categorization task illustrated in Fig. 1a. Each trial began with a fixation cross presented in the center of the screen for a random duration (500–1000 ms), which was then replaced by a face group looking straight ahead. After 800 ms, all faces averted their pupils toward left or right (all in the same direction in each trial) for another 500 ms, followed by a target item displayed in the same or the opposite direction as the gaze of the group. The participant was required to indicate the type of target, by pressing the “C” key for “kitchen” items and the “B” key for “office” items, respectively. If no response was made after 2000 ms, the trial was coded as a missing response and the next trial was presented. The interval between trials was randomly set between 1500 and 2000 ms. Both the accuracy and response time (RT) were recorded. Participants were informed that all faces were irrelevant and to be ignored.

The target item was displayed either in the direction the group gazed (the Predictive-valid group) or in the opposite direction (the Predictive-invalid group). At the beginning of the experiment, the Predictive-valid group was randomly selected to include 4 male and 4 female groups from the 16 face groups. The Predictive-invalid group then included the remaining eight groups for each participant to minimize the influence of the intrinsic trustworthiness of the faces themselves.

Each of the 16 face groups was presented in 12 trials, resulting in a total of 192 randomly presented trials. All trials were equally divided to the Predictive-valid and Predictive-invalid groups. To ensure adequate rest, this task was divided into four blocks with a 2-minute break between blocks. Before the formal task, there were at least 20 practice trials to help participants understand the instructions.

### Face-choice task and survey

As illustrated in Fig. 1b, the second part of experiment was a surprise face-choice task. For each trial, two face groups, one each from the Predictive-valid and Predictive-invalid groups, were vertically aligned in pairs. Participants were asked to choose the more trustworthy group by pressing U or J on the keyboard. No time limit was set for the response. Eight face group pairs were compared and participants’ choices were recorded.

After all trials were finished, participants were asked to complete a survey, reporting whether they indeed followed the instructions, any strategy they adopted during the experiment, and making a guess as to the experimental objective. Anyone who answered affirmatively to these questions was excluded from further analysis.

## Data analysis

Both accuracy and RT were recorded in the object categorization task. Repeated-measures ANOVAs for accuracy and RT were conducted to analyze the gaze-cueing effect in the collective, with the Predictive-validity of the group gazes (Predictive-valid vs. Predictive-invalid) as the variable. For the face-choice task, the probability of selecting the Predictive-valid group as the trustworthy one was measured by a one-sample *t*-test with a test value of 50% (chance level).

## Results

One participant reported the awareness of gaze predictability for targets and made a correct guess as to the experimental objective in the survey, so we excluded her data from further analysis. The mean accuracy in object categorization task for all participants was 97.09% ± 0.33% [Mean ± S.E.M.]. There was no main effect of Predictive-validity on accuracy [*F*(1, 28) = 0.112, *p* = 0.740, *η*_*p*_^2^ = 0.004], suggesting that the task was quite simple to complete. As illustrated in Fig. 1c, RT results yielded significant main effect for Predictive-validity [*F*(1, 28) = 9.23, *p* = 0.005, *η*_*p*_^2^ = 0.248], manifesting as a faster response for the Predictive-valid group [641.46 ± 17.03 ms] than Predictive-invalid group [659.99 ± 19.27 ms]. As for face-choice task, participants were more likely to choose the Predictive-valid face group as the trustworthy one [65.09% ± 3.00%; *t*(28) = 5.03, *p* < 0.001, *d* = 0.934; see Fig. 1d]. The results showed a gaze-cueing effect, and also supported that participants have the capability to gain incidental trust from predictable group gaze cues.

## Experiment 2

In Experiment 1, the preference shown in choosing the Predictive-valid group as more trustworthy in the surprising face-choice task constitutes primary evidence that an involuntary processing of group gaze behavior did contribute to group trust learning. However, the alternative assumption of a sampling strategy presents a challenge to the main finding. That is, participant might respond simply by looking at and forming an impression of a single person of the group in a certain position in the object categorization task, and accordingly evaluate trustworthiness on the group by recognizing this single person. To rule out the use of such a strategy, Experiment 2 added a new trustworthy contrast condition for the surprising face-choice task, in which two individuals randomly chosen from the Predictive-valid and Predictive-invalid groups, respectively, were compared. According to the sampling strategy, as participants only process a certain person in each group with three gazes, the probability of seeing this person in the face-choice task has a theoretical value of 1/3, directly resulting in a decreased percentage of evaluations of the presented Predictive-valid gaze as more trustworthy. Otherwise, if the above percentage in the group comparison scene does not significantly differ from that in a single comparison scene, we may conclude that this sampling strategy was not involved in the tasks.

## Methods

### Participants

The same data collection rules were adopted as in Experiment 1. Another group of 28 graduate and undergraduate students (13 females; mean age 19.64 years) were enrolled in this experiment. The participants provided their written informed consent in accordance with the Declaration of Helsinki before the experiment.

### Stimuli, procedure and design

**Figure 2:**
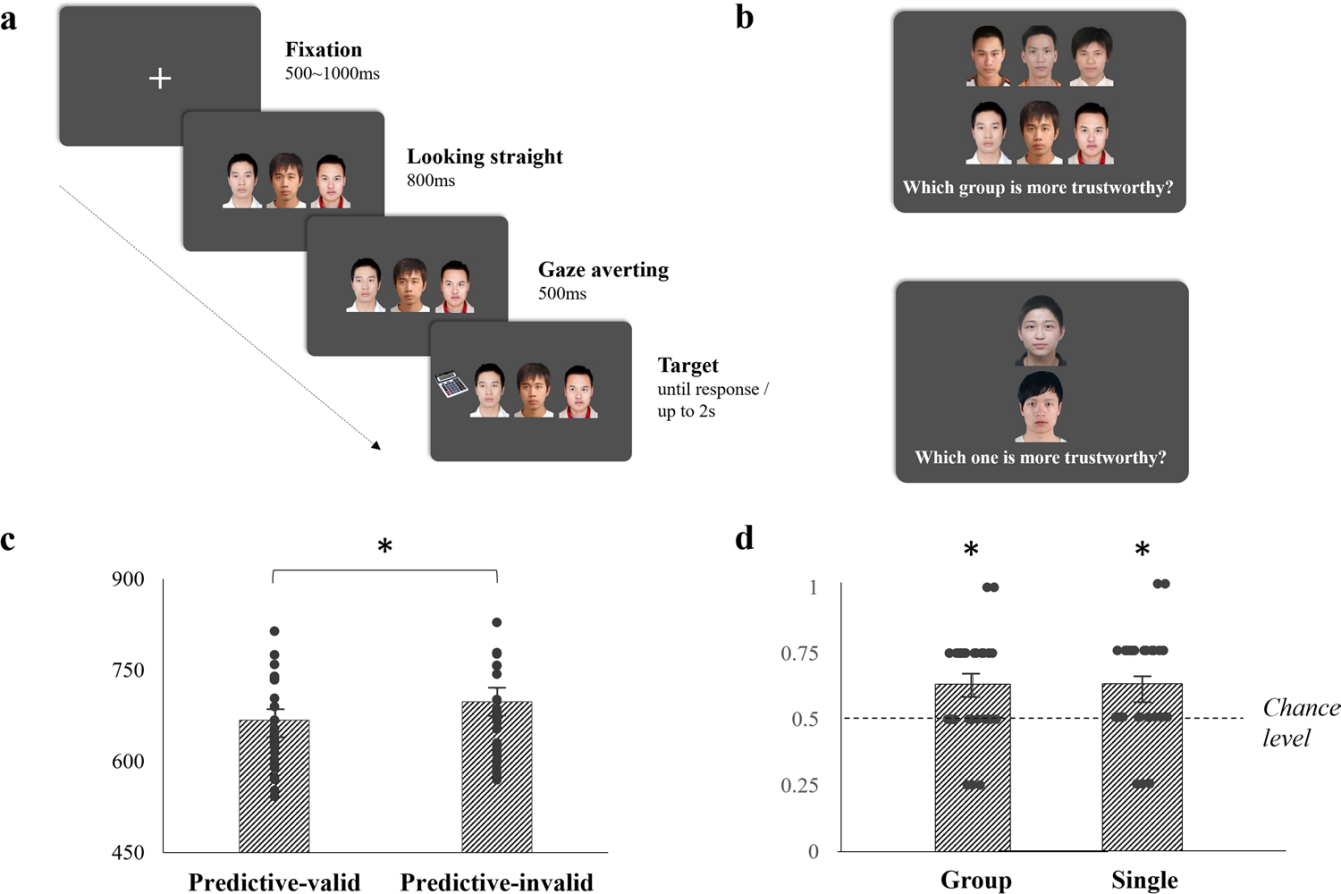
Procedure and results in Experiment 2. (**a**) An example of the trial in the object categorization task with Predictive-valid group gazes and an office item as the target; (**b**) examples of the trial to choose trustworthy group (upper panel) or single (lower panel) face(s) in the face-choice task; (**c**) RT results in the object categorization task; (**d**) results of percentage choosing the Predictive-valid group as more trustworthy, and the dashed line represents the 50% chance level. The dots in (**c**) and (**d**) are individual data points making up each group. The asterisk represents a significant difference between two corresponding conditions or from the chance level (*p* < 0.05), and the error bar represents one S.E.M.

The stimuli, general procedure, object categorization task, and survey were the same as those in Experiment 1 (see Fig. 2a).

### Face-choice task

The surprise face-choice task comprised eight trials, with number-of-people (Group vs. Single) as a new variable. In four trials (Group condition), two gaze groups, one randomly selected from the Predictive-valid or Predictive-invalid groups, were vertically aligned; in the other four trials (Single condition), two single faces, each one also randomly selected from all faces in the Predictive-valid and Predictive-invalid groups, were vertically aligned (see Fig. 2b). Participants were asked to choose the more trustworthy group/single gaze(s) by pressing U or J on the keyboard. No time limit was set for the response.

## Data analysis

In the object categorization task, repeated-measures ANOVAs for accuracy and RT were conducted to analyze the gaze-cueing effect at the collective level, with predictive-validity of the group gazes (Predictive-valid vs. Predictive-invalid) as the variable. For the face-choice task, repeated-measures ANOVA was also used to analyze the percentage selecting the Predictive-valid gaze(s) as more trustworthy, with number-of-people (Group vs. Single) as the variable. A one-sample *t*-test for each number-of-people condition was adopted with a test value of 50% (chance level).

## Results

No one answered affirmatively to the questions in the survey. The results pattern was almost the same as Experiment 1 (see Fig. 2c). In the object categorization task, there was no main effect of accuracy [*F*(1, 27) = 0.250, *p* = 0.621, *η*_*p*_^2^ = 0.009], and the mean accuracy for all participants was also very high [97.21% ± 0.36%]. Again, the main effect for RT was significant [*F*(1, 27) = 33.65, *p* < 0.001, *η*_*p*_^2^ = 0.555], showing a faster response for the Predictive-valid group [667.54 ± 20.70 ms] than the Predictive-invalid group [698.27 ± 21.29 ms].

The results in the face-choice task were illustrated in Fig. 2d. No main effect of number-of-people was found [*F*(1, 27) = 0.022, *p* = 0.882, *η*_*p*_^2^ = 0.001]. Results of *t*-tests revealed that both the probabilities of choosing Predictive-valid group [63.39% ± 3.75%; *t*(27) = 3.58, *p* = 0.001, *d* = 0.676] and single gaze [62.50% ± 3.75%; *t*(27) = 3.33, *p* = 0.002, *d* = 0.630] as more trustworthy significantly exceeded chance level. These results, especially the non-effect on number-of-people, provided supportive evidence for ruling out the sampling assumption, and gave a further verification of group trust learning from gazes.

## Experiment 3

In Experiment 1, the group gaze appeared simultaneously, as can happen in real life on occasion. However, it is not representative of the majority of real scenes. Most group interactions are gradual, in which we accumulate knowledge of the group via multiple interactions with group members (not all the members). The ability to sequentially integrate stimuli plays an important role in group perception. Thus, instead of presenting gazes in a collective simultaneously, we presented them sequentially in Experiment 3 to confirm this ability.

## Methods

### Participants

The same data collection rules were adopted as in Experiment 1. Another group of 30 graduate and undergraduate students (20 females; mean age 19.87 years) was enrolled in this experiment. The participants provided written informed consent in accordance with the Declaration of Helsinki before the experiment.

### Stimuli

Six male and six female Asian faces were selected from the face stimuli pool in Experiment 1. All faces were randomly divided into four groups (Groups A, B, C, and D). Three versions of each face were produced using Adobe Photoshop CS6, one with gaze directed straight ahead and the others averted leftward and rightward. Each face measured 2° × 2.8°, and was always displayed in the center of the screen. All groups were randomly divided into two conditions as either the Predictive-valid or Predictive-invalid group (counterbalanced among participants). The three faces in a given group were all predictive-valid or -invalid.

The target stimuli were the same as in Experiment 1.

## Procedure and design

We followed the same general procedure as in Experiment 1. Two main tasks were sequentially arranged, with mild revisions for the object categorization task and mostly the same design in the face-choice task.

### Object categorization task

**Figure 3:**
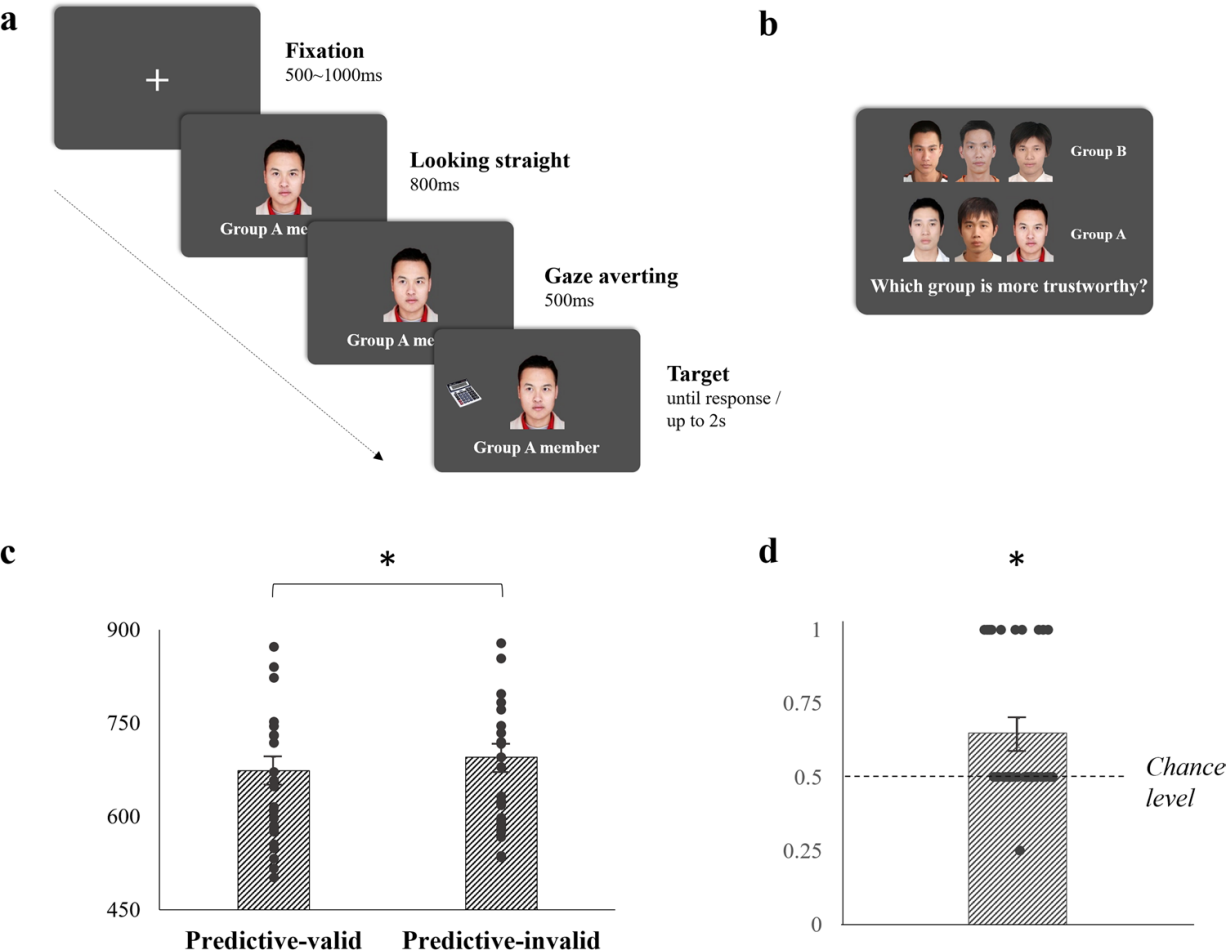
Procedure and results in Experiment 3. (**a**) An example of the trial in the object categorization task with a Predictive-valid single gaze in Group A and an office item as the target; (**b**) an example of the trial in the face-choice task; (**c**) RT results in the object categorization task; (**d**) results of percentage choosing the Predictive-valid group as more trustworthy, and the dashed line represents the 50% chance level. The dots in (**c**) and (**d**) are individual data points making up each group. The asterisk represents a significant difference between two corresponding conditions or from the chance level (*p* < 0.05), and the error bar represents one S.E.M.

Each trial began with a fixation cross in the center, which was then replaced by one face looking straight ahead and his/her member identity description below (Fig. 3a). After being presented for 800 ms, the face averted his/her pupils left or right for 500 ms, followed by a target item displayed in the same or the opposite direction as the gaze. The participant was required to indicate the type of target by pressing the “C” key for “kitchen” items and the “B” key for “office” items. The time window for response was 2000 ms, and the inter-trial interval was set randomly from 1500 to 2000 ms. Both the accuracy and RT were recorded.

The target item was always displayed in the gazing direction (for a face in the Predictive-valid group) or the opposite gazing direction (for a face in the Predictive-invalid group). Participants were told that all faces were irrelevant and to be ignored.

Each face was presented 12 times, with half of the gazes averted leftward and half rightward, resulting in a total of 144 randomly presented trials. All trials were equally divided to the Predictive-valid and Predictive-invalid groups. This task was divided into three blocks with a 2-minute break between blocks. Before the formal task, at least 20 practice trials were set to ensure participant understanding the instructions.

### Face-choice task and survey

Figure 3b shows that this task was almost the same as that in Experiment 1. The only change was that the group name was added on the right side of the group trustworthiness comparison page. The same survey was conducted as in Experiment 1.

## Data analysis

The analysis methods were the same as for Experiment 1.

## Results

No one responded affirmatively to the questions in the survey. Experiment 3 replicated the results pattern in Experiment 1 (see Figs. 3c and 3d). The accuracies were still fairly high [96.25% ± 0.49%], and the main effect was non-significant [*F*(1, 29) = 0.061, *p* = 0.807, *η*_*p*_^2^ = 0.002]. The ANOVA for RT revealed that participants had faster responses during trials with Predictive-valid gazes [673.80 ± 21.53 ms] than with Predictive-invalid gazes [695.22 ± 22.72 ms; *F*(1, 29) = 14.95, *p* = 0.001, *η*_*p*_^2^ = 0.34]. In the face-choice task, results of *t*-tests demonstrated the probability of choosing Predictive-valid group as more trustworthy significantly exceed chance level [65.83% ± 4.56%; *t*(29) = 3.47, *p* = 0.002, *d* = 0.634]. By using a sequential presenting way, Experiment 3 replicated both the gaze-cueing effect and trust learning results from predictable group gaze cues in Experiment 1.

## Discussion

As the first opportunity to obtain evidence for trust learning from group gaze behaviors, the current study conducted three experiments and obtained uniform results. Besides a solid gaze cueing effect in the collective situation, the groups in which gazes always correctly predicted the appearance position of the targets were found to be more trustworthy than the others in all experiments, although participants were told to ignore the face and gaze information throughout the experiment. An involuntary and routinized integrating process of group gaze information was accordingly revealed. Collectively, these results extend past investigations of group impression formation from static visual information to dynamic one (gaze cueing behavior).

Experiment 1 used a presenting pattern in which three gaze stimuli in a group were simultaneously displayed and participants had a short time window to process all the gazes. The preliminary results showed that individuals are able to process multiple gaze cues and transfer their attention accordingly, although gaze information is not evidently relevant to current tasks. We suggest that attention shift induced by gaze direction happens involuntarily, replicating previous findings on collective gazes^36,37^. On the other hand, and more importantly, the gazing behaviors and face information can be bound together to form a group representation, and an incidental sense of trust was gained accordingly based on the predictive validity of group gazes. The results of the face-choice task indicated that the Predictive-valid gaze group was considered more trustworthy than the Predictive-invalid gaze group, thus implying that humans are capable of integrating information as well as forming a group impression by extracting their social significance. Moreover, the current finding extends this trust learning effect from a single person to a collective scene.

Experiment 2 was designed to examine the involvement of a sampling strategy that might explain the results in Experiment 1. As the primary concern, the distinction of task performance between two number-of-people conditions was non-significant; that is to say, based on the random selection manipulation in the face-choice task, the current results constitute affirmative evidence that participants generated a trust impression for all gaze groups, confirming a group gaze perception and trust learning process. Therefore, the assumption of a sampling strategy can be ruled out. Beyond that, the instructions in all experiments emphasized that face information was unrelated to the task, and participants were told nothing about the surprise face-choice task before they finished the object categorization task. Logically speaking, participants did not need to adopt a strategy to process these “irrelevant”-gazed faces, let alone voluntarily form a group impression.

Experiment 3 used a sequential presentation of group gazes, thus examining the ability to update and organize multi-gaze information. This ability is of prime importance in real life, since different aspects of the information about a collective are more likely to be collected and updated via repeated contacts. The results verified that sequentially presented gazes with the same group label would be efficiently represented as an integration and their identical predictive validity would add specific characteristics to this representation, to finally yield a sense of trust of the whole group. To summarize, a group impression could also be formed via continuous accumulation of the behaviors of the individuals of the group.

The proposed process of group trust learning from gazes has theoretical implications in two respects. First, the use of gaze stimuli, which is a kind of interactive visual cue, constituted an initial attempt to investigate group trust learning. Group trust, especially group impressions, is the subject of a long history of research in sociology and organizational behavior^40,43^. From previous studies with semantic group descriptions, we have learned a great deal about group phenomena, including stereotyping, social categorization, and group prejudice^44^. However, the validity of these linguistic stimuli has been questioned since most of our interactions with groups start with perceptual, nonverbal processes^45^. More recently, a growing literature on group impressions has focused on the influence of sensory and perceptual cues such as gender and facial expression proportions^32,46,47^. This study is the first to adopt dynamic gaze cues to advance from the static cues used in previous studies. The current significant results confirmed distinguishable impacts on group perception and trust learning from gaze cues with different dynamic information. Second, unlike recent group impression studies with clear task goals about processing on visual cues^32,46^, the current research went to great lengths to avoid participants’ speculations on the experimental purpose and voluntary processing of gazed faces. One action is to inform participants of only the object categorization task at the beginning of the experiment and set the face-choice task as a surprise task; another is to emphasize categorizing targets regardless of gazed faces. The surveys conducted after the experiments show that most participants followed instructions and remained unaware of the gaze predictability for the target position (only one participant answered affirmatively). In line with previous findings on group trait inference using semantic stimuli^28,40^, the present results demonstrated a robust, involuntary ability to generate a complex group impression of trust from group cues.

Besides this overall effect, current research on group trust learning from gaze information also inspired several more nuanced questions, most of which had not be raised at the individual level. First, certain group properties were found to affect the impression formation for a group. Taking the entitativity of a group as an example ^40,48^, research showed that the high-entitativity (vs. low-entitativity) group facilitated group perception and trait inference, so an analogical effect might exist in group trust impression formation. Future research on this assumption could shed light on the boundary of the trust-learning effect from group gazes. Second, the current findings provide an important first step toward understanding the processing efficiency of group trust learning. Differentiating the efficiencies between group and individual trust learning is a very important point for further investigating underlying mechanism of group trust learning from gazes. Third, regarding the subsequent influence of gaze information, recent studies found a gaze leading effect^49,50^, in which the observer would show a processing bias for a person on the screen when their saccades were mirrored by his (her) gaze. For instance, people would prefer^51^ or develop a better memory^52^ of a face that followed their saccades. We believe that highlighting this new interaction pattern is especially important for exploring group trust-learning behaviors. Finally, there may be cultural differences in the trust learning effect from gaze group. Unlike westerners, East Asians have a long history with a collectivist culture, making them more sensitive to group perception and cognition^53,54^. Hence, it seems plausible to posit a more involuntary processing of group information in East Asians than westerners.

To conclude, although previous studies found that people can gain a sense of trust from another’s gaze, they mainly focused on individuals. To enrich and expand the applicability of the incidental trust learning effect from gazes, we confirmed that this phenomenon occurs at the collective level, highlighting the robustness of this effect for the first time. Regardless of the pattern of presenting gazes (simultaneously and sequentially in Experiments 1 and 3, respectively), participants were able to gain an incidental sense of trust for the collective from their consistent information of gaze directions in an involuntary manner, and this was not the result of a sampling strategy (Experiment 2), confirming that humans have an efficient capability to process gaze information.
